# Clinical validation of nursing diagnosis fatigue (00093) in women in the immediate hospital postpartum period

**DOI:** 10.1590/1980-220X-REEUSP-2021-0530en

**Published:** 2022-06-29

**Authors:** Bruna Valentina Zuchatti, Raisa Camilo Ferreira, Elaine Ribeiro, Erika Christiane Marocco Duran

**Affiliations:** 1 Universidade Estadual de Campinas, Faculdade de Enfermagem, Campinas, SP, Brazil.

**Keywords:** Fatigue, Nursing Diagnosis, Nursing Process, Postpartum Period, Validation Studies, Fatiga, Diagnóstico de Enfermería, Proceso de Enfermería, Período Posparto, Estudio de Validación, Fadiga, Diagnóstico de Enfermagem, Processo de Enfermagem, Período Pós-Parto, Estudo de Validação

## Abstract

**Objective:**

To perform clinical validation of nursing diagnosis fatigue (00093) in women in the immediate postpartum hospital.

**Method:**

This is a methodological and clinical validation study of the diagnostic accuracy of nursing diagnosis fatigue (00093) components in women in the immediate postpartum hospital period. Data were collected with women in the immediate postpartum hospital. Sensitivity and specificity accuracy measures, positive and negative predictive values of the aforementioned nursing diagnosis were investigated.

**Results:**

The sample consisted of 153 women in the immediate postpartum period hospitalized in the rooming-in ward, with a mean age of 27 years, whose defining characteristic with statistical significance was tiredness.

**Conclusion:**

The defining characteristics of tiredness and pain and related factor sleep deprivation presented high prevalence and high values of fatigue (00093) accuracy measures, being validated. Thus, it is believed that this study will contribute to clinical practice nurses in the correct identification of postpartum hospital fatigue, a frequent phenomenon in this population that causes much damage throughout the puerperium period. Additionally, it will corroborate the immediate conduct aiming at good outcome.

## RESUMO

**Objetivo:**

Realizar a validação clínica do diagnóstico de enfermagem fadiga (00093) em mulheres no pós-parto hospitalar imediato.

**Método:**

Trata-se de estudo metodológico, do tipo validação clínica, de acurácia diagnóstica dos componentes do diagnóstico de enfermagem fadiga (00093) em mulheres no pós-parto hospitalar imediato. A coleta de dados foi realizada junto a mulheres no pós-parto imediato hospitalar. Foram investigadas as medidas de acurácias de sensibilidade e especificidade, valores preditivos positivos e negativos do referido diagnóstico de enfermagem.

**Resultados:**

A amostra foi composta por 153 mulheres no pós-parto imediato internadas na enfermaria de alojamento conjunto, com idade média de 27 anos, cuja característica definidora com significância estatística foi cansaço.

**Conclusão:**

As características definidoras cansaço e dor e o fator relacionado privação de sono apresentaram alta prevalência e altos valores de medidas de acurácia para fadiga (00093), sendo validadas. Assim, acredita-se que este estudo contribuirá para os enfermeiros da prática clínica na identificação correta da fadiga pós-parto hospitalar, um fenômeno frequente nesta população que causa muitos prejuízos em todo período do puerpério. Além disso, corroborará na condução imediata visando bom desfecho.

## RESUMEN

**Objetivo:**

Realizar la validación clínica del diagnóstico de enfermería fatiga (00093) en mujeres en el posparto inmediato hospitalario.

**Método:**

Se trata de un estudio metodológico, de tipo validación clínica, de precisión diagnóstica de los componentes del diagnóstico de enfermería fatiga (00093) en mujeres en el puerperio hospitalario inmediato. La recolección de datos se realizó con mujeres en el hospital de posparto inmediato. Se investigaron medidas de precisión de sensibilidad y especificidad, valores predictivos positivo y negativo del referido diagnóstico de enfermería.

**Resultados:**

La muestra estuvo constituida por 153 mujeres en puerperio inmediato hospitalizadas en sala de alojamiento conjunto, con una edad promedio de 27 años, cuya característica definidora con significación estadística fue la fatiga.

**Conclusión:**

Las características definitorias de cansancio y dolor y el factor relacionado con la privación del sueño tuvieron una alta prevalencia y altos valores de precisión de las medidas de fatiga (00093), en proceso de validación. Así, se cree que este estudio contribuirá a los enfermeros de la práctica clínica en la correcta identificación de la fatiga posparto hospitalaria, un fenómeno frecuente en esta población que causa mucho daño a lo largo del puerperio. Además, se corroborará en la conducción inmediata apuntando a un buen desenlace.

## INTRODUCTION

The postpartum period is a phase in which transformations occur in a woman’s life, which are associated with physical and psychological changes. This period of time elapses from placental childbirth until the maternal reproductive organs return to the pre-pregnancy state. This period is marked, in addition to the physical pregnancy regression, by the exercise of motherhood, being characterized by behavioral, hormonal and social adaptations, including adapting to the new routine and the demands of a new infant, marking a phase of greater vulnerability to health problems for women^(1)^.

Thus, this group of women needs special attention from health institutions and professionals, requiring attention and commitment in the care provided to the dyad and the family^(1)^.

In addition, actions aimed at newborns (NB) and mothers are expected, so that there is a serial assessment regarding the presence and severity of physical and emotional changes, as well as the bond establishment and maintenance and care for a new member of the family nucleus.

It should be noted that adaptations will also depend on the circumstances related to the type of childbirth and the environment in which it occurred^(1–2)^.

For efficient care, the puerperium is subdivided into four phases: immediate postpartum, mediate postpartum, late postpartum and remote postpartum. The immediate period begins after the end of childbirth, called the fourth period of childbirth, in which prevalent complications are severe, related to hemorrhage, and its consequences include the period of up to 2 hours postpartum. The mediate period goes from the 2^nd^ hour to the 10^th^ postpartum day, when regression of genital organs is evident, the ligation is scarce and yellowish and lactation is fully installed. The late period lasts from day 11 to day 42 postpartum. The remote period follows from the 42^nd^ day onwards, with variable duration and with the presence or absence of lactation. Menstruation returns to non-breastfeeding women characterized by the presence of ovulation^(1,2)^.

During the postpartum period, some indicators may be observed, such as headache, bleeding, problems in breastfeeding, such as nipple fissures, sleep disorders, anxiety, fear, distress, lack of energy, prolonged sadness, low self-esteem, feelings of guilt, lack of interest in the infant, changes in appetite and fatigue^(3–6)^.

These transformations, especially the fact of becoming a mother along with hormonal issues, have many influences on women’s life and health. These postpartum stressors can lead to increased physical, emotional and mental illness, with a long-term impact on women’s and their families’ well-being^(3–6)^.

Sleep changes are also frequent problems. Some studies show that 60% to 65% of postpartum women sleep very little, i.e., less than six hours, intervening in the dynamics of maternal routine and causing fatigue^(5,6)^.

It is important to point out that studies on fatigue in the postpartum period are scarce, even though this phenomenon is frequent and has been related to several changes that are harmful to maternal life and well-being. That said, it should be noted that there is a need for further studies and qualified interventions, aiming at improving women’s physical, mental and emotional health during the immediate postpartum period^(4,6)^.

In this context, the postpartum period requires an accurate care plan subsidized by the nursing process (NP), which is configured as a methodological instrument that uses the nurses’ own body of knowledge in search of the expected results^(7)^.

The NP is a problem-solving method and determines aspects of human responses, directing the care plan. It is regulated by the Federal Nursing Council (COFEN) Resolution 358/2009, organized into five interrelated and interdependent stages: nursing history; nursing diagnosis (ND); nursing planning; nursing implementation; and nursing assessment^(7)^.

The ND can be defined as a clinical judgment about the human responses of an individual, family or community to life processes, health problems or even a vulnerability, being essential to carry out a care plan that aims at patients’ clinical improvement^(8)^.

In order to favor the diagnostic process, there is the classification of nursing diagnoses by NANDA International (NANDA-I), which names and groups the phenomena for which the nurse is responsible^(8)^. This standardized language system has in each ND its theoretical definition, defining characteristics (DC), consisting of the observable or communicable signs and symptoms that support their presence, as well as related or risk factors (RF) and associated conditions (AC)^(8)^.

Among the NDs proposed by NANDA-I, there is ND fatigue (00093), which has by definition the “an overwhelming sustained sense of exhaustion and decreased capacity for physical and mental work at usual level”. Such ND was inserted in the NANDA-I version in 1988, revised in 1998 and 2017, belongs to Domain 4 Activity/Rest, Class 3 Energy Balance^(8)^.

Presents 16 DC: alteration in concentration; alteration in libido; apathy; increase in rest requirement; increase in physical symptoms; tiredness; impaired ability to maintain usual routines; impaired ability to maintain usual physical activity; guilt about difficulty maintaining responsibilities; ineffective role performance; disinterest in surroundings; insufficient energy; introspection; listlessness; lethargy; and nonrestorative sleep pattern. It also presents nine RF: anxiety; increase in physical exertion; environmental barrier; depression; malnutrition; nonstimulating lifestyle; stressors; physical deconditioning; and sleep deprivation. Populations at risk are exposures to negative life events and demanding profession. Anemia, disease and pregnancy are AC^(8)^.

It is recommended that the ND and its components undergo review and validation processes for improvement. This process should be subsidized by rigorous methodologies, scientifically based, for the refinement of the set of clinical indicators, guiding the care provided. Its use by nurses in different populations is also allowed, making the ND susceptible to coverage by its refinement, improvement, theoretical-practical articulation, optimizing communication, nursing records and conducting research^(9–10)^.

Diagnostic validation is defined as a refining process in which degree of representativeness and predictive power of each ND’s constituent elements is assessed by enumerating the set of attributes present in the clinical environment, which determines the presence or absence of a diagnosis in a given context or scenario^(9,10)^.

Thus, the present study aimed to perform clinical validation of ND fatigue (00093) in women in the immediate postpartum hospital.

## METHOD

### Design of Study

This is a methodological and clinical validation study of diagnostic accuracy of ND fatigue (00093) components in women in the immediate postpartum hospital.

### Population

The population consisted of puerperal women who were hospitalized in the rooming-in of a high-complexity hospital, a reference in women’s health in the countryside of the state of São Paulo, a region that encompasses 42 municipalities and almost five million people.

### Local

The study site was a high-complexity university hospital in the countryside of the state of São Paulo, and in 2018, 2,862 births took place at this location, which has a rooming-in unit with approximately 24 beds.

### Sample Definition

The sample size was for convenience^(11)^, and the sample consisted of 153 puerperal women. Considering the monthly average of approximately 100 deliveries and multiplying by the total number of months of data collection (4 months), a total of 400 postpartum women were obtained, of which 153 participated in the research (n = 38.25%). It is emphasized that, in March of that year, only in the initial week there was data collection, since the world was devastated by the COVID-19 pandemic, and the collection, suspended. It is also noteworthy that neonates who required hospitalization in neonatal intensive care or were in isolation due to flu syndrome were not considered for data collection.

### Selection Criteria

Inclusion criteria comprised women who had normal vaginal childbirth, vaginal childbirth with forceps and cesarean section. Exclusion criteria comprised pain reported as a limiting factor, that is, those who reported experiencing unbearable pain, the worst pain of their lives, postpartum hemorrhage, and those who were unable to respond to the questionnaire.

### Data Collection

The study took place in a rooming-in unit, and the collection was carried out by the researcher between December 2019 and March 2020. To carry out the collection, the terms were read and explained to participants, as well as the doubts were resolved. After the proper clarifications, the Informed Consent Form (ICF) was signed in cases of participation agreement.

There is no consensus in literature on data collection. People who make the diagnostic inference are considered, after qualification and training, an imperfect gold standard. Therefore, regardless of whether she is the lead researcher or diagnostic professionals trained by her, there will be bias. In this case, what was the culmination for the choice of data collection and the diagnostic inference process to be carried out by the researcher was the fact of her expertise with the research phenomenon in this population and her accurate diagnostic reasoning process.

The researcher applied data collection instruments, namely: nursing history, which contained sociodemographic and clinical data collected by consulting the medical records; physical exam; direct observation and anamnesis; gynecological and obstetric history; information about infant; food; sleep habits (sleep quality was questioned – good, regular or poor; amount of sleep – insufficient and sufficient; whether a person considered sleep to be restorative or not; whether they are able to take advantage of NB’s sleep time to sleep; whether they had unintentional awakenings); hygiene; and binomial adaptation.

As pain was frequently reported by puerperal women, a question about the level of pain was added (unbearable – the worst pain in life and a limiting factor; bearable – pain present, but not limiting; mild pain present in a low amount and not limiting and absent pain). We chose not to characterize pain by scales, as there is no recommendation in the hospital about its use, and there was a confusion in the puerperal women when answering. Pain quality, pain irradiation, duration of improvement and worsening factors were also questioned.

To determine the presence of ND fatigue (00093), the defining characteristics and RF were listed together with their conceptual (DC) and operational (OD) definitions, elaborated in the integrative review stage. Therefore, based on the information obtained during the interview and the diagnostic definition, the researcher marked the presence or absence of each ND component.

It was decided to include, in the research, puerperal women with more than 48 hours of postpartum, disregarding the prepartum hospitalization time. Data collection took place in the patient’s room, and lasted on average 20 minutes.

### Data Analysis and Treatment

The collected data were stored in spreadsheets and analyzed by Microsoft Excel, version 2019. Descriptive and inferential statistics were performed, supported by statistical software Statistical Analysis System (SAS), version 9.4, and Statistical Package for Social Science (SPSS), version 22.0, to investigate the accuracy measures: sensitivity, specificity, positive predictive value (PPV) and negative predictive value (NPV)^(8)^.

Sensitivity represents the probability that the ND is present when the indicator or contributing factor is present. Specificity represents the probability that the ND is absent when the clinical indicator or contributing factor is absent. PPV is the percentage of individuals who have the clinical indicator or contributing factor and the ND is present and the NPV is the percentage of individuals who have the clinical indicator or contributing factor absent and the ND is absent^(8)^.

The hypotheses tested in this research were the correlation of ND fatigue (00093) with the population studied and its elements with statistically significant accuracy values that correlate with the presence or absence of the ND.

Thus, ND fatigue (00093) elements with a value above 0.500 were considered validated because they presented good discrimination power.

The other analysis that was performed is the association between sleep hours and fatigue. It was expected to observe a higher proportion of participants with fatigue among those with a lower number of hours of sleep. In this case, the null hypothesis can be written as: there is no evidence of association, considering a significance level of 5% between the variables hours of sleep and fatigue. The alternative hypothesis can be written as: there is evidence of association, considering a significance level of 5% between variables sleep hours and fatigue. For this, Pearson’s chi-square tests were performed.

### Ethical Aspects

This study followed the guidelines of Resolution 466/2012 of the Brazilian National Health Council (CNS – *Conselho Nacional de Saúde*) and was approved by the Research Ethics Committee of *Universidade Estadual de Campinas*, on October 23, 2019, under Opinion 3,656,905. After modifications requested, a new Opinion was approved, under number 4,035,696 of May 19, 2020, and only after the search began. Participants who showed acceptance to participate, read and signed the ICF in two copies, with one copy delivered to the study participant.

## RESULTS

The sample consisted of 153 women in the immediate postpartum period hospitalized in rooming-in, with a mean age of 27 years and standard deviation of 6.62, ranging from 14 to 43 years. Data collection occurred after 48 hours of childbirth. Table 1 presents the sample characterization data.

**Table 1.** Characterization of the sample of participants – Campinas, São Paulo, 2020, n = 153.

	n	%
**Age**		
14–20 years	26	17.00
21–30 years	77	50.30
31–40 years	47	30.70
41–43 years	3	2.00
**Marital status**		
Single	29	19.00
Married	43	28.10
Stable union	81	52.90
**Childbirth route**		
Vaginal	89	58.20
Caesarean section	58	37.90
Forceps	6	3.90
**Postpartum hospitalization period (hours)**		
24	44	28.70
48	37	24.20
72	72	47.00
**Injuries**		
Abdominal incisions	58	37.90
Perineal lacerations	54	35.20
Episiotomy	12	0.70

ND fatigue (00093) was present in 55.55% (n = 85), and the frequency of DC and RF was described in Table 2. It should be noted that other DC were identified during data collection, which were called additional.

**Table 2.** Distribution of defining characteristics and related factors of nursing diagnosis fatigue (00093) in women in the immediate postpartum hospital period – Campinas, São Paulo, 2020 (n = 153).

Variables	Presence		Absence		Nursing diagnosis present and defining characteristics present	
	N	%	N	%	N = 85	%
**Defining characteristics**						
Alteration in concentration	0	0	153	100.00	0	0
Alteration in libido	0	0	153	100.00	0	0
Apathy	1	0.7	152	99.30	1	1.18
Increase in rest requirement	28	18.3	125	81.70	28	32.94
Increase in physical symptoms	14	9.2	139	90.85	14	16.47
Tiredness	70	45.8	83	54.25	70	82.35
Impaired ability to maintain usual routines	1	0.7	152	99.30	1	1.18
Impaired ability to maintain usual physical activity	0	0	153	100.00	0	0
Guilt about difficulty maintaining responsibilities	0	0	0	0	0	0
Ineffective role performance	0	0	0	0	0	0
Disinterest in surroundings	0	0	0	0	0	0
Insufficient energy	21	13.7	132	86.24	21	24.71
Listlessness	36	23.5	117	76.47	36	42.35
Introspection	3	2.0	150	98.04	3	3.53
Lethargy	0	0	0	0	0	0
Nonrestorative sleep pattern	31	20	122	79.74	31	36.47
*Physical discomfort	26	16.3	127	83.01	18	21.18
*Pain	67	43.8	86	56.21	43	50.60
**Related factors**						
Anxiety	40	26.1	113	73.86	40	47.01
Increase in physical exertion	7	4.6	146	95.42	7	8.24
Environmental barrier	0	0	153	100.00	0	0
Depression	1	0.7	152	99.30	1	1.18
Malnutrition	0	0	153	100.00	0	0
Nonstimulating lifestyle	0	0	153	100.00	0	0
Stressors	3	2.0	151	98.69	3	3.53
Physical deconditioning	1	0	152	99.30	1	1.18
Sleep deprivation	71	46.4	82	53.59	71	83.53

*Additional defining characteristic.

It is emphasized that each puerperal woman could present more than one DC and/or RF. Moreover, the correlation between sleep hours and the occurrence of ND fatigue (00093) was investigated.

It was noticed that 64.00% of women who slept up to 3 hours presented ND fatigue (00093), and 39.60% who slept more than three hours also presented the aforementioned ND. Furthermore, a p-value of 0.0039 was obtained, being significant.

Accuracy measures were also applied to the RF of said ND, as they are factors that contribute to the presence or absence of a ND. Table 3 presents these indicators.

**Table 3.** Accuracy measures of defining characteristics and related factors of nursing diagnosis fatigue (00093) in women in the immediate postpartum hospital period – Campinas, São Paulo, 2020.

Defining characteristics	S	SP	PPV	NPV
Alteration in concentration	0	1.00	–	0.4444
Alteration in libido	0	1.00	–	0.4444
Apathy	0.0118	1.00	1.00	0.4474
Increase in rest requirement	0.3294	1.00	1.00	0.5440
Increase in physical symptoms	0.1647	1.00	1.00	0.4892
**Tiredness**	**0.8235**	**1.00**	**1.00**	**0.8193**
Impaired ability to maintain usual routines	0.0118	1.00	1.00	0.4474
Impaired ability to maintain usual physical activity	0	1.00	–	0.4444
Guilt about difficulty maintaining responsibilities	0	1.00	–	0.4444
Ineffective role performance	0	1.00	–	0.4444
Disinterest in surroundings	0	1.00	–	0.4444
Insufficient energy	0.2471	1.00	1.00	0.5152
Listlessness	0.4235	1.00	1.00	0.5812
Introspection	0.0353	1.00	1.00	0.4533
Lethargy	0	1.00	–	0.4444
Nonrestorative sleep pattern	0.3647	1.00	1.00	0.5574
*Physical discomfort	0.2118	0.8824	0.6923	0.4724
*Pain	**0.5059**	**0.6471**	**0.6418**	**0.5116**
**Related factors**				
Anxiety	0.4706	1.00	1.00	0.6018
Increase in physical exertion	0.0824	1.00	1.00	0.4658
Environmental barrier	0	1.00	–	0.4444
Depression	0.0118	1.00	1.00	0.4474
Malnutrition	0	1.00	–	0.4444
Nonstimulating lifestyle	0	1.00	–	0.4444
Stressors	0.0353	1.00	1.00	0.4533
Physical deconditioning	0.0118	1.00	1.00	0.4474
**Sleep deprivation**	**0.8353**	**1.00**	**1.00**	**0.8293**

_*Additional defining characteristic; S – sensitivity; SP – specificity; PPV – positive predictive value; NPV – negative predictive value._

It is evident that the absence of DC nonrestorative sleep pattern and increase in rest requirement is good for determining the absence of ND.

Additional DC pain was associated with women who underwent cesarean section and presented pain in the surgical incision (37.90%), those who had pain in the perineum region due to lacerations and/or episiotomy (35.20%) and those who had back pain due to analgesia (0.70%).

## DISCUSSION

It is notelike that there was a prevalence of young and primiparous puerperal women in this study.

In a study that aimed to observe the prevalence of maternal fatigue and its associated factors during the first six months after childbirth, it was observed that primiparity and younger maternal age are correlated with the incidence of the fatigue phenomenon, when compared with older or multiparous puerperal women^(12)^. There was also a relationship between the number of hours spent and the presence of ND fatigue (00093), in which puerperal women who slept up to three hours had said ND. This information is important for managing and controlling the number of rest hours.

In another study that sought to investigate the associations between sleep characteristics and fatigue, it was observed that a fragmented and poor quality sleep may be associated with a high prevalence of fatigue^(13)^.

DC tiredness showed greater predicting power than ND fatigue (00093). These results are similar to a study that assessed the course of postpartum fatigue, reinforcing that tiredness is the most relevant indicator for the occurrence of fatigue^(13)^.

It should be noted that tiredness and fatigue are not synonymous, as fatigue can be defined as a more severe and prolonged physical and mental sensation, and it is not so easily relieved, and tiredness can be defined as a physical sensation, which is a clinical indicator of fatigue^(12)^.

DC pain is a predictor of said ND fatigue (00093) in this population. These results are similar to a survey that compared postpartum fatigue in women who underwent vaginal and cesarean childbirth, concluding that patients who underwent interventions such as cesarean sections, episiotomies or lacerations had pain and, consequently, fatigue^(12,14,15)^.

The absence of DC apathy, increase in physical symptoms, impaired ability to maintain usual routines, insufficient energy, introspection and the additional physical discomfort are good for determining the absence of ND fatigue (00093), because when they were not observed, the ND was absent. Although these indicators are absent in the immediate postpartum period, they may appear in the late and/or remote puerperium.

It is noteworthy that DC listlessness, nonrestorative sleep pattern, increase in rest requirement were present and, even though they are not considered predictors of the aforementioned ND, they establish a correlation with each other and with women in this period. Some studies that assessed postpartum fatigue and sleep disorder showed that women in this period have fragmented sleep due to infant care and breastfeeding. Thus, these DC nonrestorative sleep pattern and increase in rest requirement are implicit in tiredness, and it is proposed to exclude these DC for this population^(14,15,16,17)^.

RF sleep deprivation was validated and can be considered the factor that most contributes to ND fatigue (00093) in women in the immediate postpartum hospital, that is, it is the most characteristic for this ND in the population studied. Some studies report that sleep disorders and sleep deprivation are associated with the establishment of maternal routine and breastfeeding, and this reduces sleeping hours and worsens the quality and amount of sleep^(13–18)^. Thus, there is a need for greater attention from health professionals as well as from the maternal support network.

The absence of RF increase in physical exertion, depression, stressors and physical deconditioning contributed to the absence of ND in women in the immediate postpartum hospital. When they were absent, the ND was not identified. Although it is an expected response, management and adaptation is needed, as it was also found that, when examining the associations between sleep and fatigue during hospitalization after an infant’s birth, it is found that sleep deprivation is a common event^(14)^.

It is suggested that anxiety, presented in this population, be addressed as a human response, i.e., identified as an interventionable ND and not only as a RF^(12)^.

We also emphasize that elements that respectively presented NPV and PPV values >0.5 are good for indicating the probability of presence and the sensitivity and accuracy measure. Therefore, DC insufficient energy (0.5152), listlessness (0.5812), nonrestorative sleep pattern (0.5574) and RF anxiety (0.6018) were not mentioned.

It should be emphasized that ND fatigue (00093) was present in women in the immediate postpartum period. Thus, it is suggested to manage this human response. It is also evident that fatigue encompasses a negative feeling, not being so easily resolved, and once installed and not managed correctly, it will be liable to damages to the mother’s health and dyad maintenance^(12,18)^.

## CONCLUSION

Fatigue is part of the process of adaptation of women in the postpartum period. Regarding clinical indicators, DC tiredness, DC additional pain and RF sleep deprivation showed high prevalence and high values of accuracy measures for ND fatigue (00093), considered validated.

Thus, this innovative and enriching study suggests the addition of DC pain, the exclusion of DC nonrestorative sleep pattern and increase in rest requirement and RF anxiety and depression in this population. ND puerperal fatigue is also suggested.

Therefore, this study may contribute to the clinical practice of nurses for the correct identification of ND fatigue in women in the immediate postpartum hospital. It is a frequent phenomenon, with significant physical and mental repercussions in the aforementioned period, which can be managed through nursing interventions, and tiredness, also expected in the physiological and mental adaptation phase, is physiological and reversible.

## ASSOCIATE EDITOR

Marcia Regina Cubas
